# Supplementary material for: Hydrogel tapes for fault-tolerant strong wet adhesion
Source: Nat Commun. 2021 Dec 9;12:7156. doi: 10.1038/s41467-021-27529-5 (PMC8660897; doi:10.1038/s41467-021-27529-5)
Supplement: Supplementary file 1 — Supplementary Information [file 41467_2021_27529_MOESM1_ESM.pdf]

Supplementary Information for

Hydrogel tapes for fault-tolerant strong wet adhesion

Bin Xue[†, 1], Jie Gu[†, 1], Lan Li[†, 2], Wenting Yu[1], Sheng Yin[1], Meng Qin[1], Qing Jiang[2, *], Wei Wang[1, 3, *] and Yi Cao[1, 3, 4, 5, *]

[1] *Collaborative Innovation Center of Advanced Microstructures, National Laboratory of Solid State Microstructure, Key Laboratory of Intelligent Optical Sensing and Manipulation, Ministry of Education, Department of Physics, Nanjing University, Nanjing 210093, China*
[2] *State Key Laboratory of Pharmaceutical Biotechnology, Department of Sports Medicine and Adult Reconstructive Surgery, Drum Tower Hospital affiliated to Medical School of Nanjing University, Nanjing, 210008, China.*
[3] *Institute for Brain Sciences, Nanjing University, Nanjing, 210093, China.*
[4] *Chemistry and Biomedicine innovation center, Nanjing University, Nanjing, 210093, China.*
[5] *Wenzhou Institute, University of Chinese Academy of Sciences, Wenzhou 325001, China*

*Correspondence to: caoyi@nju.edu.cn; wangwei@nju.edu.cn; qingj@nju.edu.cn

† These authors contributed equally to this work.

This file includes:

Supplementary Materials and Methods
Supplementary Figures 1 to 28

Table of contents

Supplementary Materials and Methods 3
Materials 3
Synthesis of polyacrylic acid (PAA) 3
Synthesis of alginate-dopa 3
Oxidization of N-acetyldopamine and alginate-dopa 4
High-performance liquid chromatography (HPLC) 4
Swelling ratio and solid content measurements 5
Scanning electron microscopy (SEM) images 5
Sealing of the lung under the cyclical insufflation 5
Burst pressure evaluation 6
In vitro biocompatibility 6
Supplementary Figures 7
Supplementary Figure 1 7
Supplementary Figure 2 7
Supplementary Figure 3 8
Supplementary Figure 4 9
Supplementary Figure 5 9
Supplementary Figure 6 10
Supplementary Figure 7 10
Supplementary Figure 8 11
Supplementary Figure 9 11
Supplementary Figure 10 12
Supplementary Figure 11 12
Supplementary Figure 12 13
Supplementary Figure 13 13
Supplementary Figure 14 14
Supplementary Figure 15 14
Supplementary Figure 16 15
Supplementary Figure 17 16
Supplementary Figure 18 16
Supplementary Figure 19 17
Supplementary Figure 20 18
Supplementary Figure 21 18
Supplementary Figure 22 19
Supplementary Figure 23 20
Supplementary Figure 24 21
Supplementary Figure 25 21
Supplementary Figure 26 22
Supplementary Figure 27 23
Supplementary Figure 28 24

Supplementary Materials and Methods

Materials

N-Hydroxy succinimide (NHS), 1-(3-dimethylaminopropyl)-3-ethylcarbodiimide hydrochloride (EDC), dihydroxyphenylalanine (dopa) and bovine serum albumin (BSA) were purchased from Shanghai Aladdin Biochemical Technology Co., Ltd. Acrylic acid was purchased from Aladdin Industrial Corporation (Shanghai, China), and sodium alginate (10 kDa) was purchased from Beijing J&K Scientific Co., Ltd. Zoletil was purchased from Virbac (S.A., France). Eight-week-old male Sprague-Dawley rats were obtained from Nanjing Medical University. New Zealand rabbits and Bama mini pigs were obtained from Anlimo (Yizheng, China). The fibrin-based bio-glue was purchased from SHRAAS (Shanghai, China). The cyanoacrylate glue was purchased from Compant (Beijing, China). ddH_2O was produced by a Milli-Q® integral water purification system (Merck KGaA, Germany). The cell line and associated culture reagents included the following: the calcein-AM and propidium iodide (PI) double staining kit (cat: KGAF001, Keygen, China), the cell culture medium AMEM and DMEM (cat: 310-010-CL and cat: 319-051-CL, Wisent, China), the culture supplement foetal bovine serum (FBS) (cat: 10091148, Gibco, USA), MC 3T3 and MEF cells (Stem Cell Bank, Chinese Academy of Sciences, China). All other chemical reagents, unless otherwise stated, were purchased from Shanghai Aladdin Biochemical Technology Co., Ltd. (China). All reagents were used without further purification.

Synthesis of polyacrylic acid (PAA)

Acrylic acid and ammonium persulfate (APS) were dissolved into ddH_2O to the concentration of 300 and 1 mg mL^{-1}, respectively. Then the polymerization was achieved under the UV irradiation for 1 hour. The product was dialyzed in ddH_2O to remove the unreacted reagents and concentrated.

Synthesis of alginate-dopa

Alginate-dopa was prepared by connecting dopa to the carboxyl group of alginate (10

kDa) in the presence of NHS and EDC. Typically, alginate and dopa were dissolved in ddH_2O to concentrations of 2.5 mM and 0.1 M, respectively. Then, EDC and NHS were added to the solution to a concentration of 0.6 M. Ascorbic acid was added into the mixture to the concentration of 0.3 M and the pH was adjusted to 7.8 using NaOH solution. Then the mixture was degassed with argon and sonicated three times (each time for 15 min) to remove dissolved oxygen. Ascorbic acid and degassing were used to prevent the oxidation of dopa. The mixture was stirred for 12 h at room temperature under the protection of argon. Finally, the unreacted reactants were removed by dialysis in ddH_2O under the protection of argon, and the product was lyophilized. The linking efficiency of alginate-dopa was confirmed with UV calibration curves (Supplementary Fig. 1).

Oxidization of N-acetyldopamine and alginate-dopa

The electro-oxidation of N-acetyldopamine or alginate-dopa was completed by a galvanic cell, as shown in Supplementary Fig. 2. In a typical electro-oxidation process, N-acetyldopamine (100 mM) or alginate-dopa (20 mg mL^{-1}) dissolved in phosphate buffer solution (PBS) (10 mM, pH=7.4) was placed at the anode. PBS (10 mM, pH=7.4) was used as the electrolyte at the cathode. The salt bridge was made of 33 w/v % agar gel containing KCl (3 M). Then, an electric potential of 5 V was applied to the system for different times. All experiments were undertaken at room temperature. The chemical oxidation of N-acetyldopamine was achieved using potassium periodate (KIO_4). Typically, KIO_4 was added into the N-acetyldopamine (100 mM) solution to the concentration of 100 mM and the solution was incubated for different times (1 or 24 h).

High-performance liquid chromatography (HPLC)

The electro-oxidized and chemically oxidized products of N-acetyldopamine were analysed with high-performance liquid spectroscopy (HPLC) and mass spectrometry. HPLC analysis was performed on a ThermoScientific U3000 system at 280 nm with a GE SOURCETM 5RPC ST 4.6/150 column. The mobile phases were as follows:

solvent A: 0.1% TFA in H_2O and solvent B: 0.1% TFA in CH_3CN. The gradient program was as follows: 0 to 20 min, 95% A to 5% A; 20 to 22 min, 5% A; 22 to 25 min, 5% to 95% A; 25 to 26 min, 95% A. All the samples were dissolved in ddH_2O.

Swelling ratio and solid content measurements

For the swelling experiments, the volume of the hydrogel after the gelation using EDC/NHS was recorded as V_1. Then, the hydrogel was immersed in 50 times PBS solutions (10 mM, pH=7.5) for 24 hours at room temperature to reach swelling equilibrium, and the volume was recorded as V_2. The swelling ratio (Ɛ) was calculated by the equation $\varepsilon = V_2/V_1$. For the solid content measurements, the hydrogel samples stored in the mixture of alcohol (75%) and ddH_2O (25%) were weighed, and the wet weight was recorded as W_1. Then, the hydrogel samples were dried, and the weight was recorded again as W_2. The solid content (ς) was calculated as $\varsigma = W_2/W_1 \times 100\%$.

Scanning electron microscopy (SEM) images

Scanning electron microscopy (SEM) images were obtained using a Quanta scanning electron microscope (Quata 200, FEI) at 20 kV. The hydrogels were dialysed in ddH_2O water for 24 h to remove the unbound salts and lyophilized material prior to measurement. The porcine skin before and after adhesions were dried in air. Then, the samples were sputter-coated with platinum and imaged with SEM.

Sealing of the lung under the cyclical insufflation

For the sealing of a lung under the cyclical insufflation, the contraction and relaxation of the lung was driven by a ventilator (MEDUMAT Standard², WEINMANN, Germany) at the mode of Intermittent Positive Pressure Ventilation (IPPV). The hydrogel tape is colored with a dark food dye for visualization. The tidal volume (V_t), frequency, maximum pressure (p_{Max}), and positive end expiratory pressure (PEEP) were first set as 275 mL, 13 breaths per min, 15 mBar, and 5 mBar, respectively. Then an injury (0.5×1.5 cm) was created to the lung. After sealing the lung using an Electro-Ox

hydrogel tape (diameter of 4.0 cm), the V_t and p_{Max} were changed to 600 mL and 40 mBar to increase the pressure applied to the lung. The cyclical insufflation lasted for 8 h.

Burst pressure evaluation

For the evaluation of burst pressures, the stomach or lung tissue with a thickness between 3-5 mm were cut off from the porcine organs. The tissues were fixed on a pumping chamber and a penetrating defect (diameter of 4 mm) was created at the center of the tissue. A hydrogel tape (10 mm×10 mm) was applied to the defect for 15 s (short-term sealing) or 24 h (long-term sealing). The pressure was increased by pumping PBS into the chamber at the speed of 2 mL min^{-1} with a syringe pump (WZS-50F6, Smiths Medical, China). The burst pressure during the test was recorded using a digital piezometer (SW-512C, SNDWAY, China).

In vitro biocompatibility

Mouse embryo osteoblast precursor (MC3T3) cells were cultivated in AMEM and mouse embryonic fibroblast (MEF) cells were cultivated in DMEM. 10% foetal bovine serum and 1% penicillin & streptomycin were added to the medium. All the cells used in cell viability testing were before 5 passages.

To assess the cell viability of cells living on gel, 100 μL gel was prepared in each well of opaque-walled multiwall plates before collecting cells, and cells were digested from culture dishes. Then, the cells were seeded into the wells containing hydrogel at a density of 6000 per well. Pristine DMEM was used as control. Cell incubation was performed at 37 °C and 5% CO_2. After 24 hours, the plates were equilibrated to room temperature for approximately half an hour. Then, a live/dead viability/cytotoxicity kit (Calcein-AM/PI Double Staining Kit) was used to evaluate cell viability. After washing the wells twice with PBS solution, calcein AM and propidium iodide (PI) dye solution were mixed and added to each well. Then, the plate was incubated at 37 °C for 30 min before being washed with PBS (10 mM, pH=7.4) 3 times. Finally, images were obtained

using an OLYMPUS-IX73 fluorescence microscope (OLYMPUS, USA).

Supplementary Figures

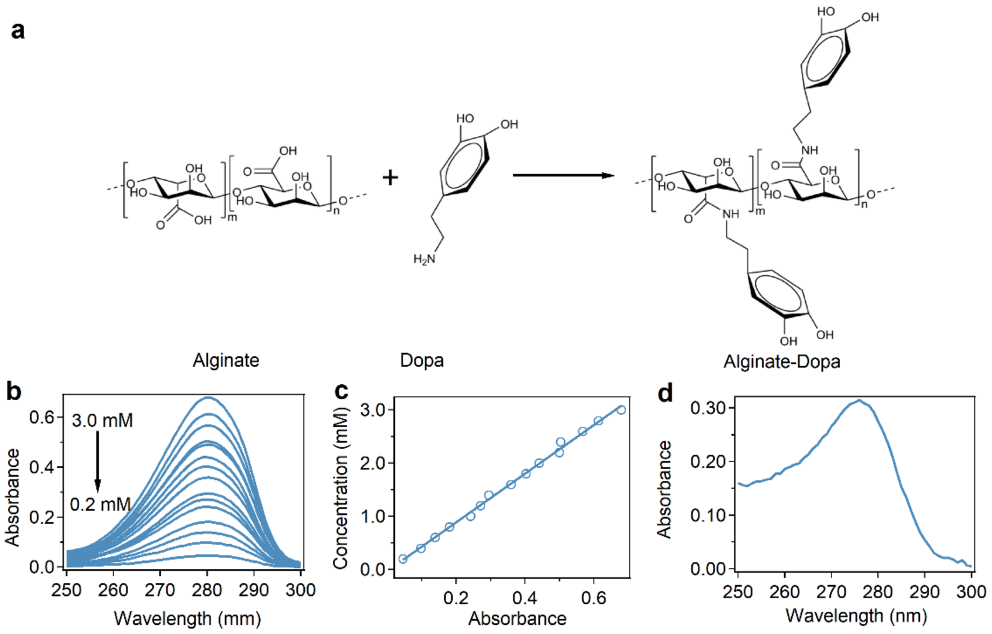

Supplementary Figure 1. Determination of the conjunction efficiency of dopa and alginate. **a**, Chemical structure of alginate and alginate-dopa. **b**, UV-vis spectra of dopa at various concentrations (0.2-3.0 mM). **c**, Calibration curve of $OD_{320\ nm}$ and dopa concentrations. **d**, UV absorbance of the alginate-dopa at the concentration of 1 mg mL^{-1}. The mass percentage of dopa in alginate-dopa is ~21%.

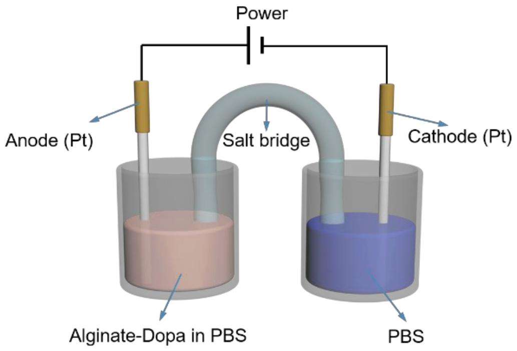

Supplementary Figure 2. Electro-oxidization of the dopa contained ploymers using the galvanic cell. N-acetyldopamine (100 mM) or alginate-dopa (20 mg mL^{-1}) dissolved in PBS (10mM, pH=7.4) was placed at the anode and a voltage of 5 V was applied to the system.

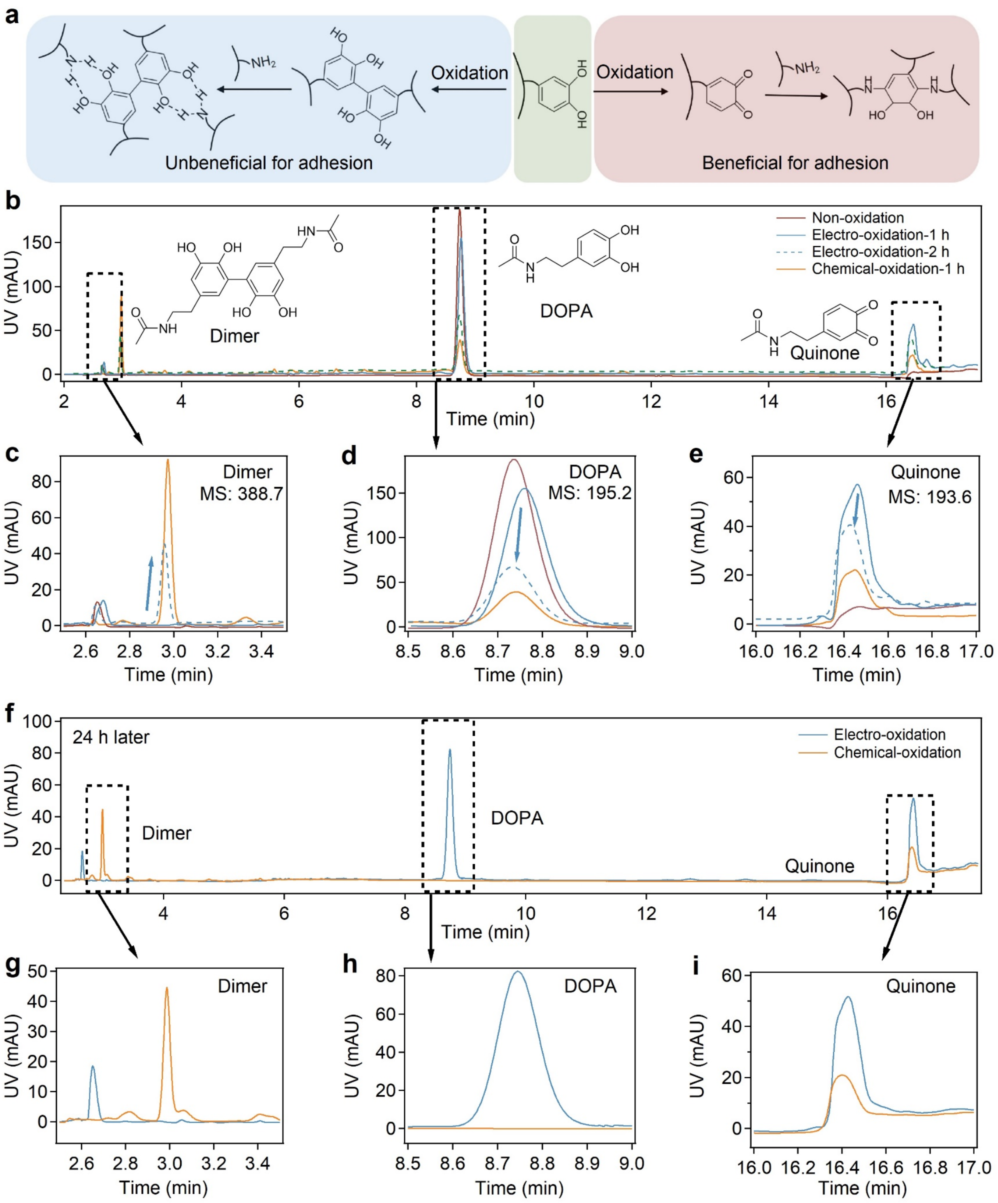

Supplementary Figure 3. Schematic and characterization of the electro-oxidation of N-acetyldopamine. N-acetyldopamine was chosen as the model molecule and oxidized using different methods to confirm the products of electro- and chemical-oxidization. **a**, Different oxidation reaction pathway of dopa. The production of dimer is useless for the surface adhension of dopa contained materials. In contrast, the production of dopaquinone would extremely benefit the tissue adhension due to the covalent junction with amino. **b**, HPLC and mass analyses of N-acetyldopamine and the electro- or chemical-oxidation products of N-acetyldopamine. In the electro-oxidation (1 h), most of the phenolic hydroxyl groups changed into quinone while

those in the chemical-oxidation (1 h) formed dimer. After further electro-oxidation of N-acetyldopamine by extending the electro-oxidation time (2 h), the quinone in the products would gradually converted to dimer due to the increased dopaquinone concentration. **c-e**, Zoomed-in HPLC curves of the dimer (**c**), dopa (**d**) and quinone (**e**) in **b**. **f**, HPLC and mass analyses of the electro- or chemical-oxidation products of N-acetyldopamine after storage for 24 h. The remianed dopa in the products of chemical-oxidation changed into dimer beacuse of the remanent oxidant while the electro-oxidized products slightly changed, indicating the stability and controllability of the electro-oxidation. **g-i**, Zoomed-in HPLC curves of the dimer (**g**), dopa (**h**) and quinone (**i**) in **f**.

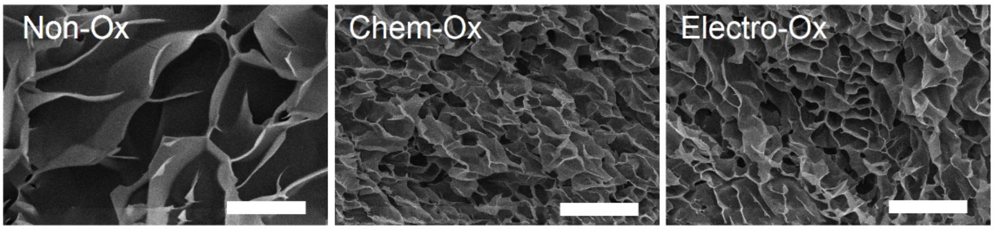

Supplementary Figure 4. SEM images of the Non-Ox, Chem-Ox and Electro-Ox hydrogels. Scale bar = 100 μm. All the experiments were repeated at least three times with similar results.

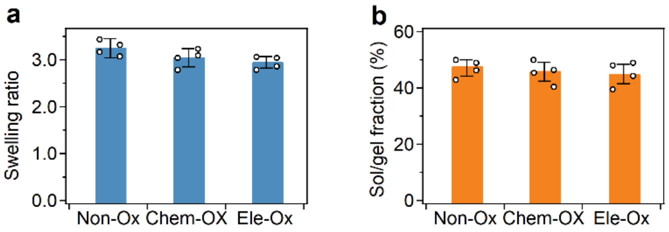

Supplementary Figure 5. Swelling ratio (a) in PBS and so/gel fraction (b) in the mixture of alcohol (75%) and ddH_2O (25%) for different hydrogel at the alginate-dopa of 10 w/v%. Values represent the mean and standard deviation (n=4 independent samples).

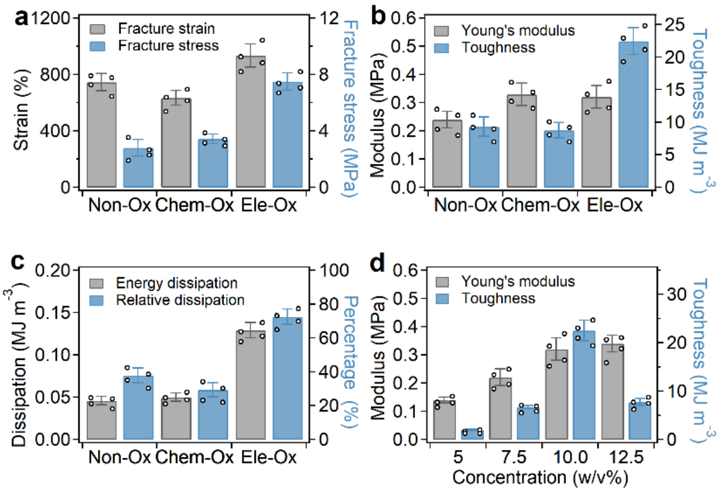

Supplementary Figure 6. Mechanical characterization of Non-Ox, Chem-Ox and Electro-Ox hydrogels. **a**, Fracture strain and fracture stress. **b**, Young's modulus and toughness. **c**, Energy dissipation and relative dissipation ratio. **d**, Young's modulus and toughness of Electro-Ox hydrogel at various mass concentration of electro-oxidized alginate-dopa. Values represent the mean and standard deviation (n=4 independent samples).

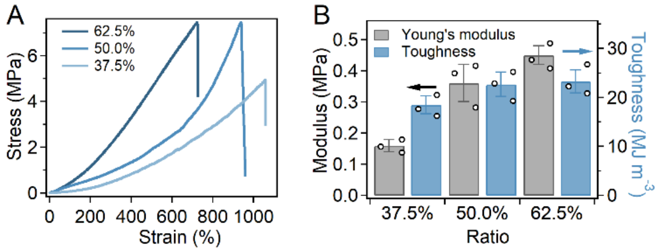

Supplementary Figure 7. Mechanical properties of the Electro-Ox hydrogel tape at different total mass concentrations. **a**, Typical stress versus stretch curves of Electro-Ox hydrogels at various total mass concentration (37.5, 50.0 and 62.5 w/v%). **b**, Summarized Young's modulus and toughness of the hydrogel tape corresponding to **a**. Values represent the mean and standard deviation (n=3 independent samples).

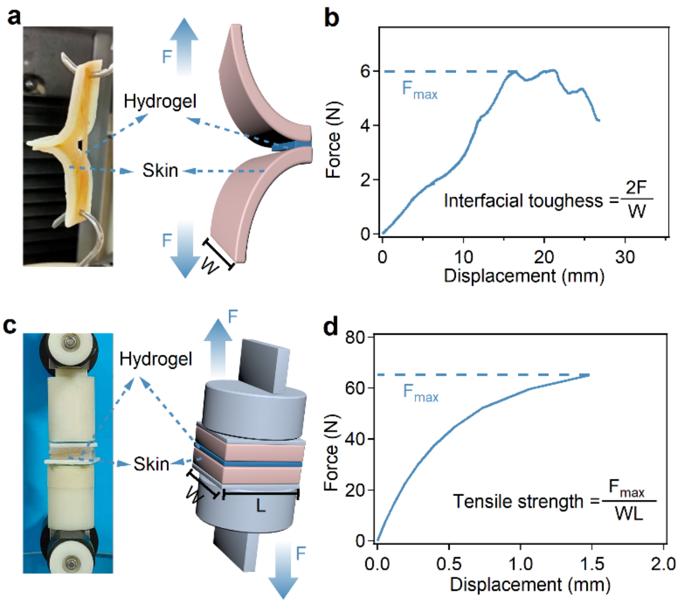

Supplementary Figure 8. Setups for mechanical testing of adhesion performance. **a**, **b**, Schematic (**a**) and typical stress-strain curves (**b**) of the interfacial toughness test. **c**, **d**, Schematic (**c**) and typical stress-strain curves (**d**) of the tensile strength test.

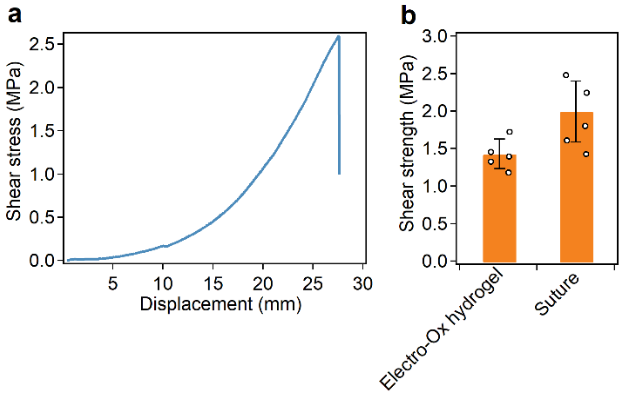

Supplementary Figure 9. Breaking strength of porcine skins jointed by traditional sutures. **a,** Typical force-displacement curve. **b**, Comparison of the breaking strength for the porcine skins joined using Electro-Ox hydrogel tapes and sutures. Values represent the mean and standard deviation (n = 5 independent samples).

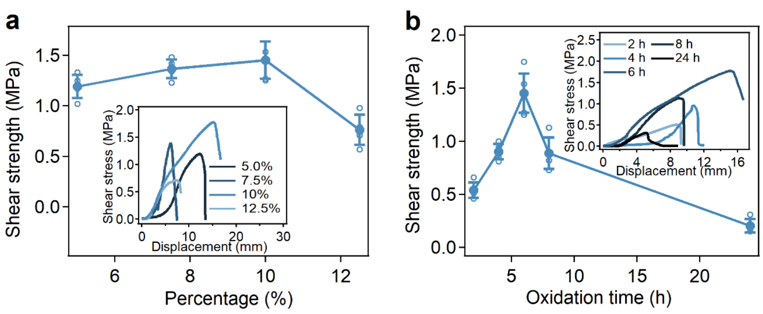

Supplementary Figure 10. Optimization of adhesion strength by varying the concentration and the electro-oxidation time of alginate-dopa. **a**, Shear strength of wet porcine skin adhered using the hydrogel tape containing various concentrations of electro-oxidized alginate-dopa (5, 7.5, 10 and 12.5 w/v%). **b**, Shear strength of wet porcine skin adhered using the hydrogel tape containing alginate-dopa electro-oxidized for different times (2, 4, 6, 8 and 24 h). Values represent the mean and standard deviation (n=4-5 independent samples).

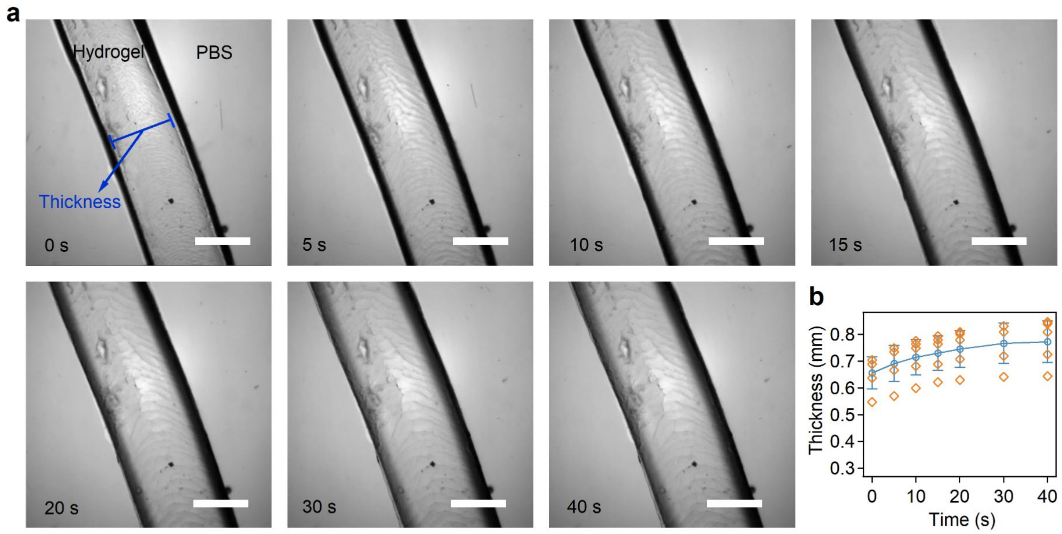

Supplementary Figure 11. Swelling of the Electro-Ox hydrogel in PBS solution (10 mM, pH=7.4). **a,** Microscopic images of the cross section of the Electro-Ox hydrogel after soaking the hydrogel in PBS solutions for different times. The experiments were repeated three times with similar results. Scale bar = 500 µm. **b,** Summarized variation of the thickness of hydrogels with time. Values represent the mean and standard deviation (n=5 independent samples).

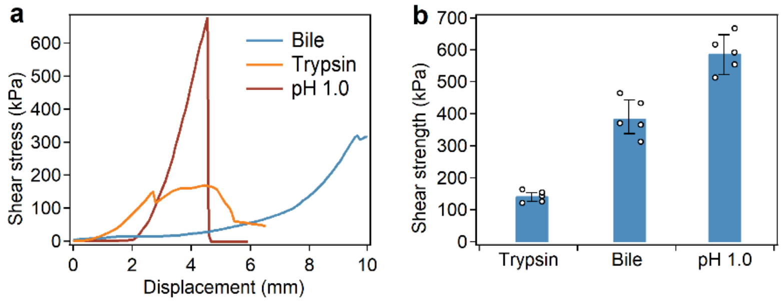

Supplementary Figure 12. Adhesion of porcine skins using the Electro-Ox hydrogel tape in the presence of bile/trypsin or a low pH solution (pH=1.0). **a**, Typical force-displacement curve of the lap shear test. **b**, Shear strength measured at low pH or with enzymes. Values represent the mean and standard deviation (n = 5 independent samples).

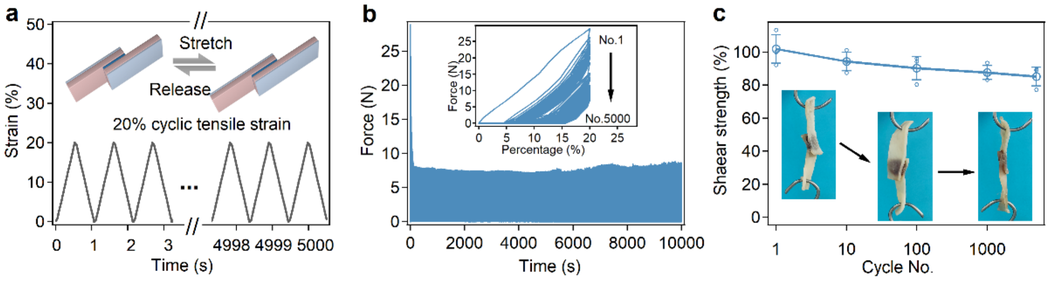

Supplementary Figure 13. Stabilities of the long-term tissue adhesion of the Electro-Ox hydrogel tapes. **a**, Cyclic stretch-relaxation at the strain of 20% was applied to the porcine skin adhered by Electro-Ox hydrogel tape. Inset corresponds to the schematic of the stretch-relaxation cycles. **b**, Typical stretch-relaxation curves versus time of the adhered porcine skins in 5000 loading cycles. Inset corresponds to the stretch-relaxation curves versus strain. The force decreased gradually due to the plastic deformation of porcine skins. **c**, Shear strength of the adhered porcine skin as a function of the cyclic number of 20% tensile strain applied to the porcine skin. Values represent the mean and standard deviation (n=3 independent samples).

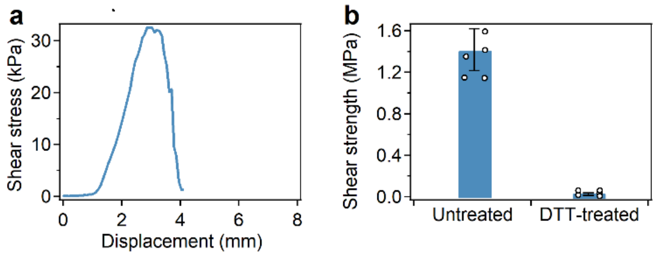

Supplementary Figure 14. Shear strength of the porcine skins adhered using the Electro-Ox hydrogel tape after being treated with DTT solutions (1 mM). **a**, Typical force-displacement curve of the lap shear test. **b**, Comparison of the shear strength of untreated and DTT-treated samples after long-term adhesion was established. Values represent the mean and standard deviation (n = 5 independent samples).

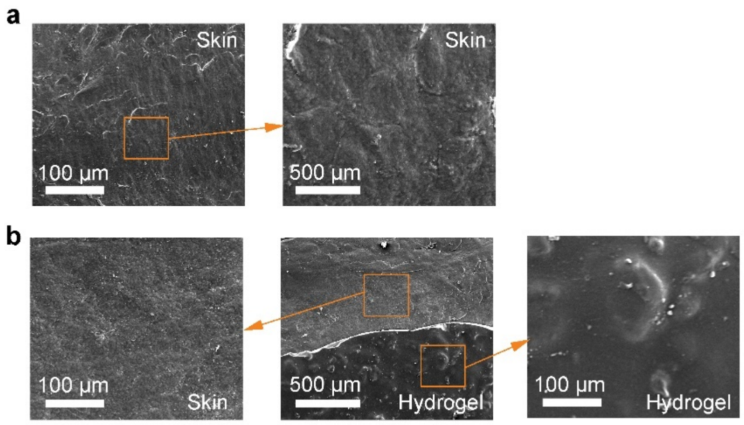

Supplementary Figure 15. SEM images of porcine skin surface. **a**, Before the adhesion of the Electro-Ox hydrogel tape. **b**, After removing of the Electro-Ox hydrogel tape. To directly compare the skin surface with and without the hydrogel tape, the hydrogel tape was only partially removed. All experiments were repeated three times with similar results.

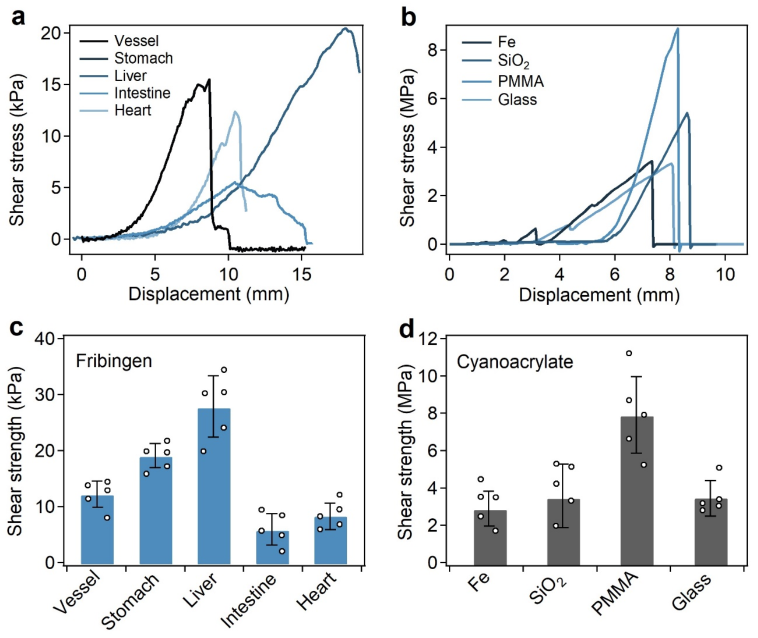

Supplementary Figure 16. Adhesion strengths of commercially available bio-adhesives. **a,** Typical force-displacement curves of the lap shear test for the adhesion of different porcine organs using the fibrinogen-based bio-adhesive. **b**, Typical force-displacement curves of the lap shear test for the adhesion of different substrates using the cyanoacrylate-based glue. **c,** Summary of the adhesion strength for different porcine organs using the fibrinogen-based bio-adhesive. **d,** Summary of the adhesion strength for different substrates using the cyanoacrylate-based glue. The glued samples were placed at room temperature for at least 24 h to ensure the adhesion strength reached the maximum. Values in **c-d** represent the mean and standard deviation (n=5 independent samples).

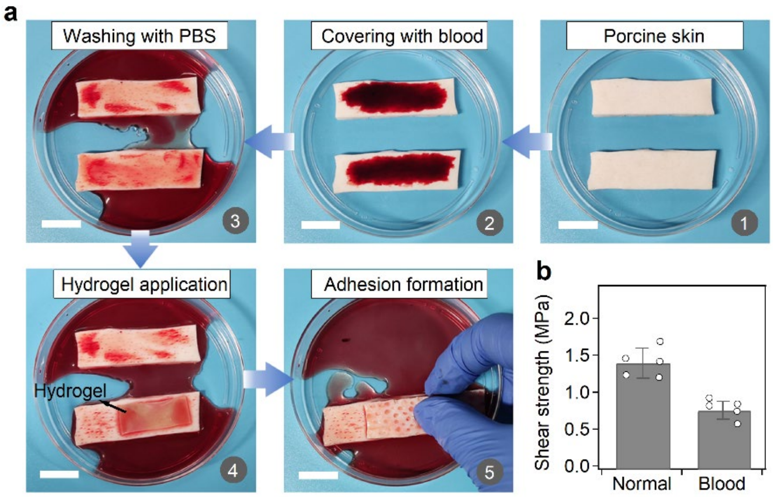

Supplementary Figure 17. Adhesion performance of Electro-Ox hydrogel tapes on blood covered porcine skins. **a**, Adhesion of porcine skin covered with blood using Electro-Ox hydrogel tape. Scale bar = 20 mm. **b**, Long-term adhesion strength between porcine skins without and with blood. Values represent the mean and standard deviation (n = 5 independent samples).

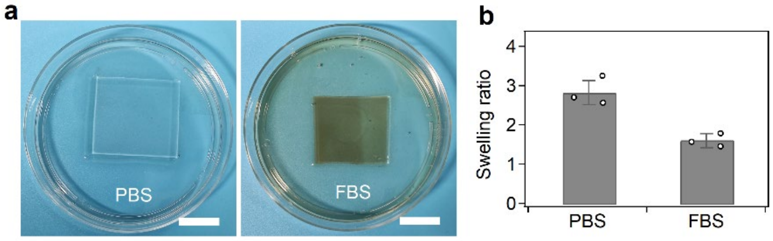

Supplementary Figure 18. Swelling of Electro-Ox hydrogels in PBS and foetal bovine serum (FBS). **a**, Images of Electro-Ox hydrogels with the same initial size after swelling in PBS and FBS for 48 hours. Scale bar = 20 mm. **b**, Swelling ratios of Electro-Ox hydrogels in PBS and FBS. Values represent the mean and standard deviation (n = 3 independent samples).

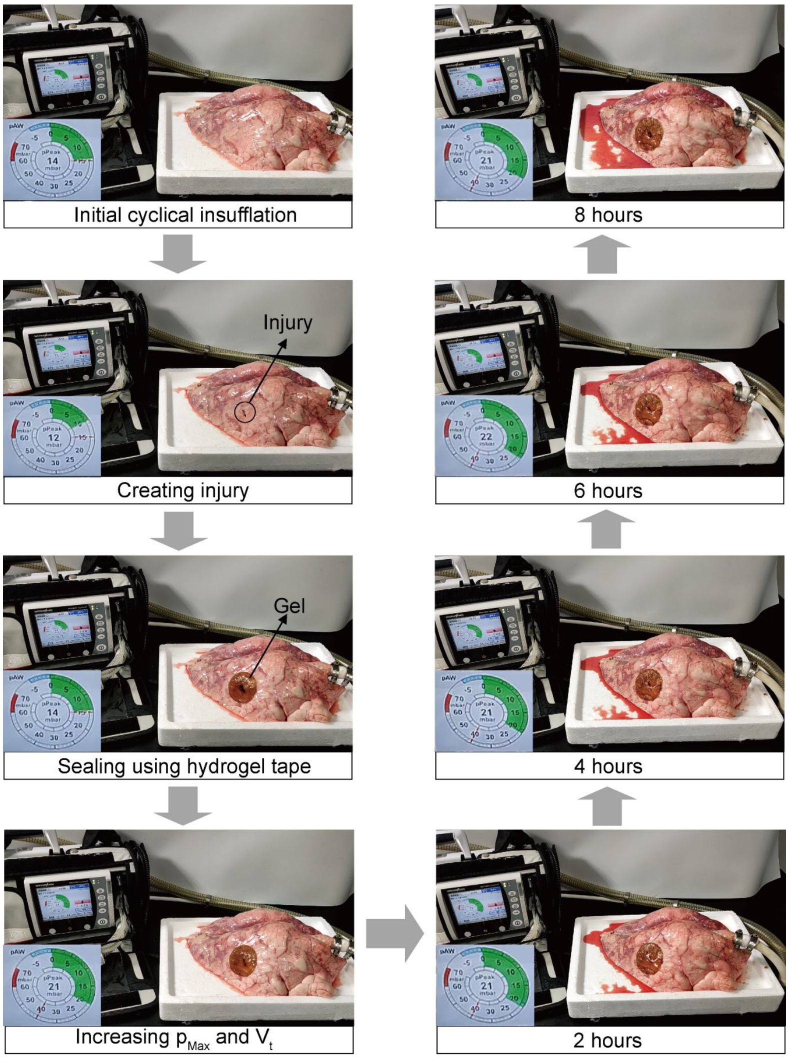

Supplementary Figure 19. Air-tight lung sealing under the cyclical insufflation using the Electro-Ox hydrogel tape. The value of p_{peak} recovered from 12 mBar of the injured lung to 14 mBar of the sealed lung, which was the same as that of the uninjured lung. After increasing the p_{Max} and V_t to 40 mBar and 600 mL, the p_{peak} increased to 21 mBar and remained stable at the range of 21-22 mBar in the following 8 h of cyclical insufflation. The lung and hydrogel were sprayed with PBS solution (10 mM, pH=7.4) to keep wet during the test.

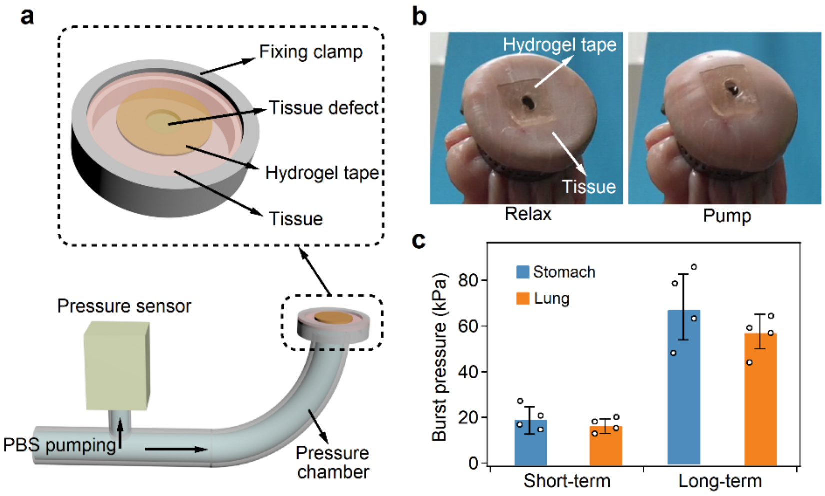

Supplementary Figure 20. Burst pressure of the porcine stomach and lung sealed by the Electro-Ox hydrogel tapes. **a**, Schematic illustration of the burst pressure measurement using the punctured porcine tissues. **b**, Typical images of the burst pressure measurements using the sealed porcine stomach as an example. **c**, Burst pressures of short-term and long-term sealing for the porcine stomach and lung. Values represent the mean and standard deviation (n = 4 independent samples).

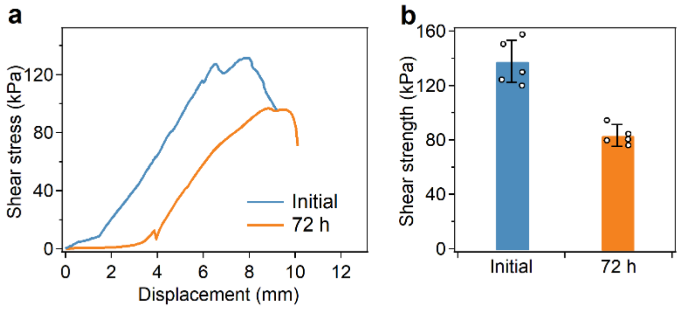

Supplementary Figure 21. Adhesion strength of the hydrogel-tape applied to the rabbit heart in vivo. **a**, Typical force-displacement curves of the lap shear test. **b**, Summary of the shear strength. The “initial” group is the shear strength after long-term adhesion was established and the “72 h” group is the shear strength after in vivo adhesion for 72 h. Values represent the mean and standard deviation (n = 5 independent samples).

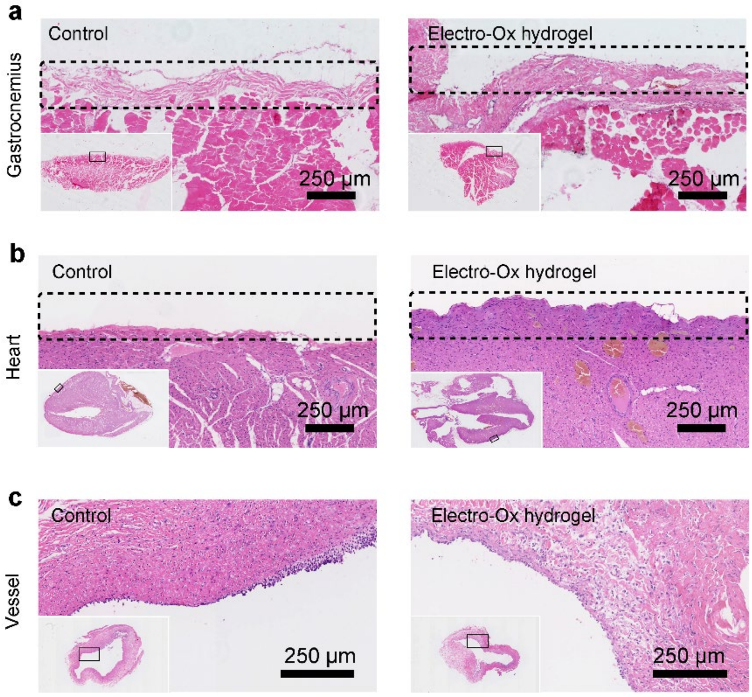

Supplementary Figure 22. Representative H&E staining of the tissues in the in vivo adhesion and haemostasis using the Electro-Ox hydrogel tapes. **a**, Representative H&E staining of rabbit gastrocnemius after in vivo adhesion with an Electro-Ox hydrogel tape for 72 h and then the hydrogel was torn off. The sham surgery was set as the control group. **b**, Representative H&E staining of rabbit heart after in vivo adhesion of Electro-Ox hydrogel tape for 72 h and then the hydrogel was torn off. The normal heart was set as the control group. **c**, Representative H&E staining of porcine vessel after in vivo adhesion of Electro-Ox hydrogel tape for 72 h and then the hydrogel was torn off. The sham surgery was set as the control group. All experiments were repeated three or four times with similar results.

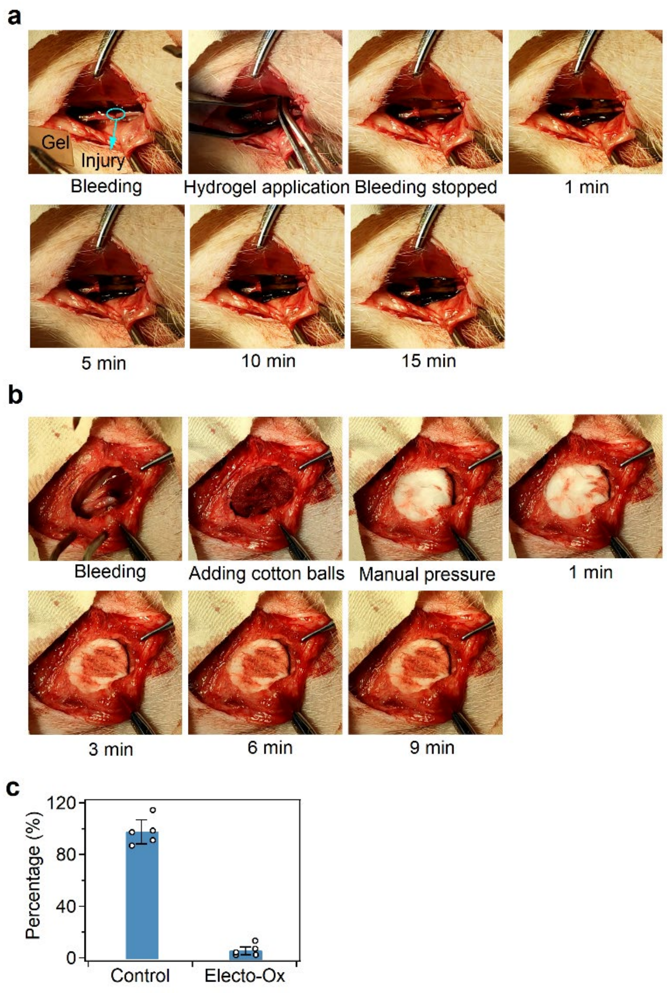

Supplementary Figure 23. Haemostasis of the vascular injury of pigs. **a**, Haemostasis of the vascular injury of pigs using Electro-Ox hydrogel tapes in vivo. **b**, Haemostasis of the vascular injury of pigs by manual pressure using medical cotton balls in vivo. **c**, Comparison of the amounts of bleeding using different methods. Values represent the mean and standard deviation (n = 5 independent experiments).

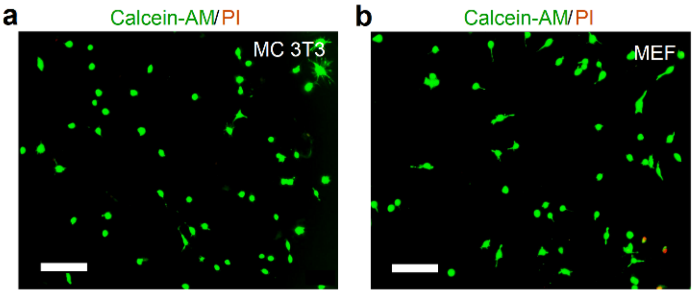

Supplementary Figure 24. Fluorescence microscope images of MC 3T3 (**a**) and MEF (**b**) cells cultured in the control medium. The living and dead cells were stained with live/dead assay (Calcein-AM/PI Double Staining Kit) after 24 h of culture. The experiments were repeated five times with similar results. Scale bar = 100 μm.

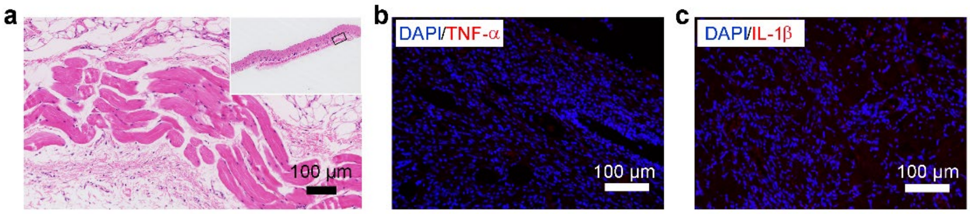

Supplementary Figure 25. H&E and inflammatory factor staining of the subcutaneous tissues from rats without surgery. **a,** Representative image of the H&E staining. **b, c,** Representative immunostaining images of TNF-α (**b**) and IL-1β (**c**) of the subcutaneous tissues from rats without surgery. Cell nuclei are indicated by DAPI (Blue). No inflammatory effect can be observed on the tissues without surgery. Scale bar=100 μm. The experiments were repeated three times with similar results.

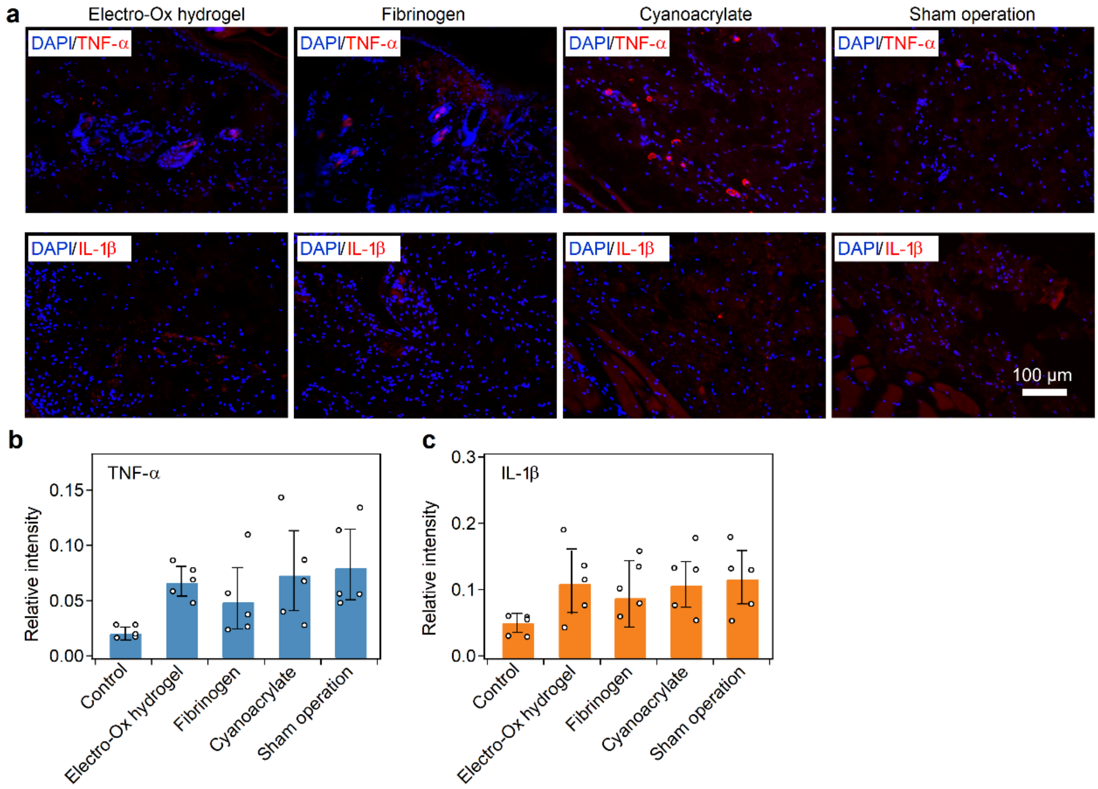

Supplementary Figure 26. Evaluation of the inflammatory responses in the first day after the subcutaneous implantation. **a,** Representative immunostaining images identified by molecular markers (TNF-α or IL-1β, colored in red). Cell nuclei are indicated by DAPI (blue). Scale bar=100 μm. **b-c,** Relative intensity of TNF-α and DAPI (b) or IL-1β and DAPI (c) in the images for various samples and the one without surgery was set as the control. Values represent the mean and the standard deviation (n = 5 biologically independent animals).

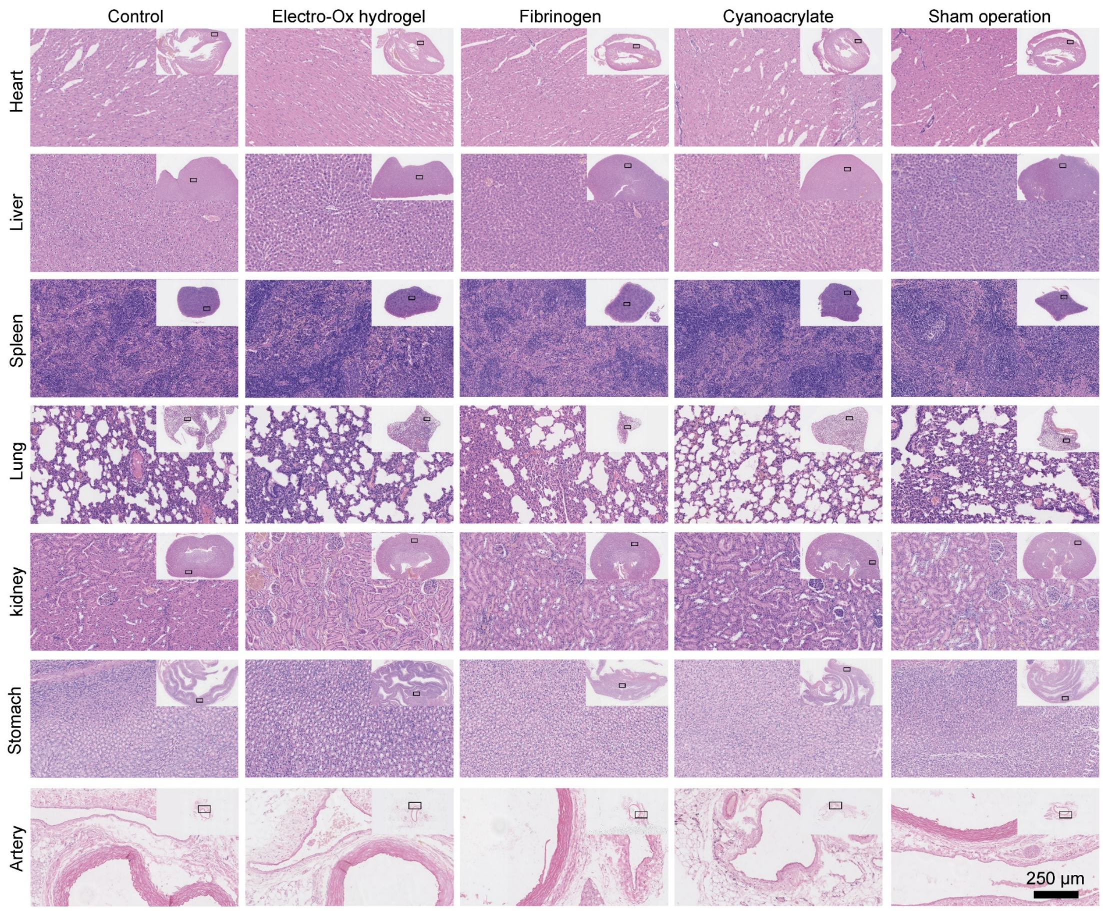

Supplementary Figure 27. Representative H&E staining of major organs (the heart, liver, spleen, lung, kidney, stomach and artery) in 1 day after subcutaneous implantation of different bio-adhesives in the backs of Sprague Dawley rats. The organs from the rats without surgery were set as the control group. All histological experiments were repeated at least three times with similar results.

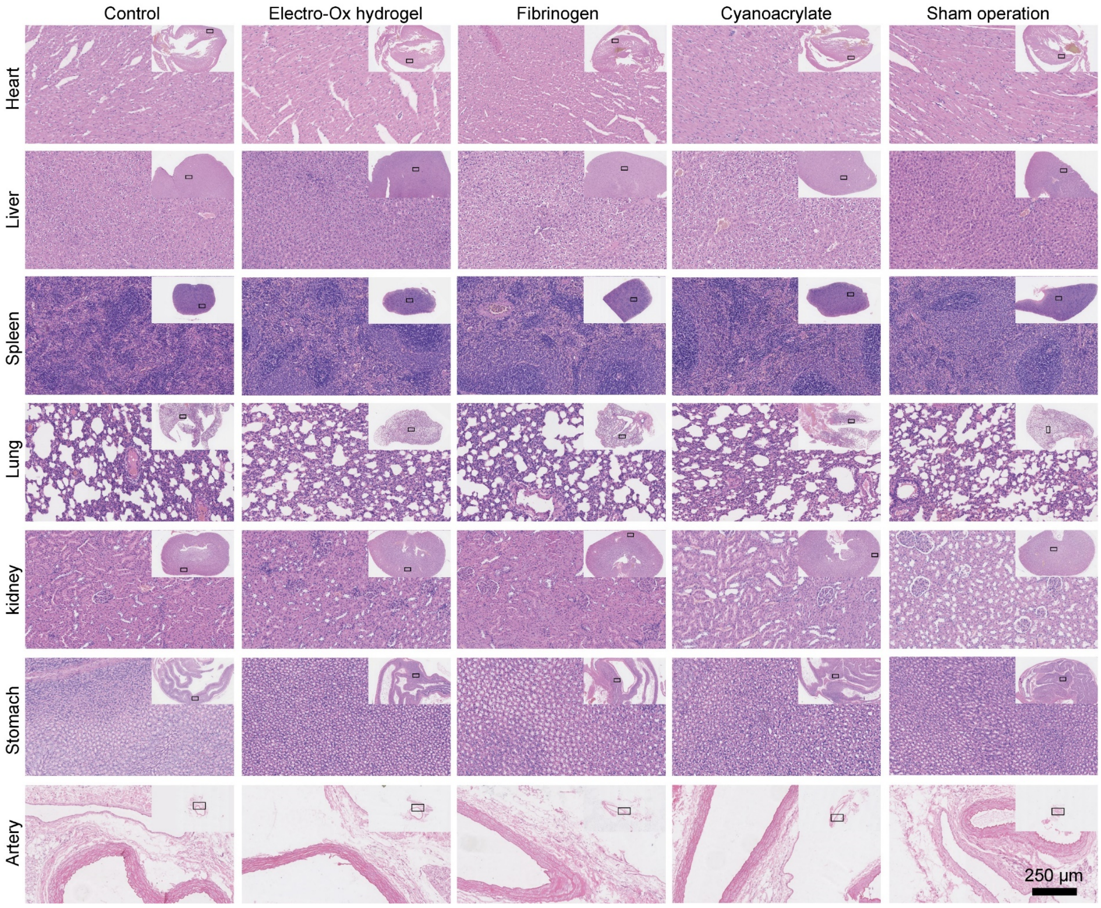

Supplementary Figure 28. Representative H&E staining of major organs (the heart, liver, spleen, lung, kidney, stomach and artery) at the time point of 14 days after subcutaneous implantation of different bio-adhesives in the backs of Sprague Dawley rats. The organs from the rats without surgery were set as the control group. All histological experiments were repeated at least three times with similar results.
