# Supplementary material for: Hydrogel tapes for fault-tolerant strong wet adhesion
Source: Nat Commun. 2021 Dec 9;12:7156. doi: 10.1038/s41467-021-27529-5 (PMC8660897; doi:10.1038/s41467-021-27529-5)
Supplement: Supplementary file 3 — Description of Additional Supplementary Files [file 41467_2021_27529_MOESM3_ESM.docx]

**Description of Additional Supplementary Files**

**Supplementary Movie 1** | Sealing of a water-leaking stomach in vitro.

**Supplementary Movie 2** | Sealing of an air-leaking lung in vitro.

**Supplementary Movie 3** | Sensor adhesion application in vitro.

**Supplementary Movie 4** | Gastrocnemius adhesion in vivo.

**Supplementary Movie 5** | Device adhesion on beating heart in vivo.

**Supplementary Movie 6** | Vessel rupture hemostasis in vivo.
