# Supplementary material for: Hydrogel tapes for fault-tolerant strong wet adhesion
Source: Nat Commun. 2021 Dec 9;12:7156. doi: 10.1038/s41467-021-27529-5 (PMC8660897; doi:10.1038/s41467-021-27529-5)
Supplement: Supplementary file 10 — Reporting Summary [file 41467_2021_27529_MOESM10_ESM.pdf]

nature portfolio

Corresponding author(s): Yi Cao
Last updated by author(s): Nov 4, 2021

Reporting Summary

Nature Portfolio wishes to improve the reproducibility of the work that we publish. This form provides structure for consistency and transparency in reporting. For further information on Nature Portfolio policies, see our Editorial Policies and the Editorial Policy Checklist.

Statistics

For all statistical analyses, confirm that the following items are present in the figure legend, table legend, main text, or Methods section.

n/a	Confirmed	
☐	☒	The exact sample size (*n*) for each experimental group/condition, given as a discrete number and unit of measurement
☐	☒	A statement on whether measurements were taken from distinct samples or whether the same sample was measured repeatedly
☐	☒	The statistical test(s) used AND whether they are one- or two-sided<br>*Only common tests should be described solely by name; describe more complex techniques in the Methods section.*
☒	☐	A description of all covariates tested
☐	☒	A description of any assumptions or corrections, such as tests of normality and adjustment for multiple comparisons
☐	☒	A full description of the statistical parameters including central tendency (e.g. means) or other basic estimates (e.g. regression coefficient) AND variation (e.g. standard deviation) or associated estimates of uncertainty (e.g. confidence intervals)
☐	☒	For null hypothesis testing, the test statistic (e.g. *F*, *t*, *r*) with confidence intervals, effect sizes, degrees of freedom and *P* value noted<br>*Give P values as exact values whenever suitable.*
☒	☐	For Bayesian analysis, information on the choice of priors and Markov chain Monte Carlo settings
☒	☐	For hierarchical and complex designs, identification of the appropriate level for tests and full reporting of outcomes
☒	☐	Estimates of effect sizes (e.g. Cohen's *d*, Pearson's *r*), indicating how they were calculated

Our web collection on statistics for biologists contains articles on many of the points above.

Software and code

Policy information about availability of computer code

Data collection	Data were collected with various laboratory instruments and equipments. Such as: tensile-compressive tester (Instron-5944, USA),high-performance liquid spectroscopy(HPLC, ThermoScientific U3000, USA), Quanta scanning electron microscope (Quata 200, FEI, USA), LCR meter (HIOKI-IM3536, Japan), digital piezometer (SW-512C, SNDWAY, China) and fluorescence microscope (OLYMPUS-IX73, USA).
Data analysis	Igor6.37， ImageJ V 1.8.0, IBM SPSS statistics 26, Microsoft Excel 2019.

For manuscripts utilizing custom algorithms or software that are central to the research but not yet described in published literature, software must be made available to editors and reviewers. We strongly encourage code deposition in a community repository (e.g. GitHub). See the Nature Portfolio guidelines for submitting code & software for further information.

Data

Policy information about availability of data

All manuscripts must include a data availability statement. This statement should provide the following information, where applicable:

- Accession codes, unique identifiers, or web links for publicly available datasets
- A description of any restrictions on data availability
- For clinical datasets or third party data, please ensure that the statement adheres to our policy

All data is available in the main text, the supplementary information and the supplementary movies. The source data underlying Figs. 2-4, Fig. 6, supplementary Fig. 1, supplementary Fig. 3, supplementary Figs. 5-14, supplementary Figs. 16-18, supplementary Figs. 20-21, supplementary Fig. 23 and supplementary Fig. 26 are provided as Source Data file. Source data are provided with this paper.

Field-specific reporting

Please select the one below that is the best fit for your research. If you are not sure, read the appropriate sections before making your selection.

☒ Life sciences ☐ Behavioural & social sciences ☐ Ecological, evolutionary & environmental sciences

For a reference copy of the document with all sections, see nature.com/documents/nr-reporting-summary-flat.pdf

Life sciences study design

All studies must disclose on these points even when the disclosure is negative.

Sample size	Sample sizes were chosen based on previously published literature and protocols in the field with a similar setup that showed statistical significance (Nature 575, 169-174 (2019)). For each experiment, we have adopted at least n=3 biological replicates to calculate the statistical value of each analysis and the exact sample sizes and statistical data for each experiment are reported in the figure legends.
Data exclusions	No data were excluded from the analyses.
Replication	All experiments were repeated at least three times with reproducibility. All attempts at replication were successful and the exact numbers are indicated in the figure legends or table for all experiments.
Randomization	Rats , rabbits and pigs were assorted randomly to cages when received from the vendor. All the tests were performed with randomly allocated experimental groups.
Blinding	The investigators were blinded to group allocation during data collection and analysis.

Reporting for specific materials, systems and methods

We require information from authors about some types of materials, experimental systems and methods used in many studies. Here, indicate whether each material, system or method listed is relevant to your study. If you are not sure if a list item applies to your research, read the appropriate section before selecting a response.

Materials & experimental systems

n/a	Involved in the study	
☒	☐	Antibodies
☐	☒	Eukaryotic cell lines
☒	☐	Palaeontology and archaeology
☐	☒	Animals and other organisms
☒	☐	Human research participants
☒	☐	Clinical data
☒	☐	Dual use research of concern

Methods

n/a	Involved in the study	
☒	☐	ChIP-seq
☒	☐	Flow cytometry
☒	☐	MRI-based neuroimaging

Eukaryotic cell lines

Policy information about cell lines

Cell line source(s)	The cell lines of MC 3T3 and MEF were purchased from Stem Cell Bank, Chinese Academy of Sciences, China.
Authentication	Authentication was performed by analysis of morphology.
Mycoplasma contamination	All cell lines tested negative for mycoplasma contamination.
Commonly misidentified lines (See ICLAC register)	No commonly misidentified lines were used in this study.

Animals and other organisms

Policy information about studies involving animals; ARRIVE guidelines recommended for reporting animal research

Laboratory animals	Eight-week-old male Sprague-Dawley rats were obtained form Nanjing Medical University. Nine-week-old female New Zealand rabbits and four-month-old male Bama mini pigs were obtained from Anlimo, Yizheng, China.
Wild animals	The study did not involve wild animals.

Field-collected samples	This study did not involve samples collected from the field.
Ethics oversight	All animal studies were carried out in compliance with the regulations and guidelines of the Ethics Committee of Drum Tower Hospital affiliated to the Medical School of Nanjing University and conducted according to the Institutional Animal Care and Use Committee (IACUC) guidelines.

Note that full information on the approval of the study protocol must also be provided in the manuscript.
